# Preoperative total cholesterol and axillary lymph node burden in breast cancer: an exploratory analysis with a preliminary nomogram

**DOI:** 10.3389/fsurg.2026.1860780

**Published:** 2026-07-01

**Authors:** Xingxing Ouyang, Lisha Wu, Yanhua Zhou

**Affiliations:** 1 Department of Breast Surgery, The Affiliated Cancer Hospital of Xiangya School of Medicine, Central South University, Hunan Cancer Hospital, Changsha, Hunan, China; 2 Department of Operation Room, The First Hospital Affiliated with Hunan Normal University, Hunan Provincial People’s Hospital, Changsha, Hunan, China; 3 Department of Hospital Infection, The Affiliated Cancer Hospital of Xiangya School of Medicine, Central South University, Hunan Cancer Hospital, Changsha, Hunan, China

**Keywords:** axillary lymph node burden, breast cancer, internal validation, nomogram, risk prediction, total cholesterol

## Abstract

**Purpose:**

High axillary lymph node (ALN) burden is an important factor in treatment planning for breast cancer. This study aimed to explore the association between preoperative serum total cholesterol (TC) and high ALN burden and to develop a preliminary risk estimation model.

**Methods:**

This retrospective study included 100 patients with primary breast cancer who underwent upfront modified radical mastectomy. High ALN burden was defined as more than four metastatic lymph nodes. Logistic regression analysis was used to assess the association between TC and nodal burden, with adjustment for tumor size. Model performance was evaluated using receiver operating characteristic analysis and bootstrap internal validation.

**Results:**

High ALN burden occurred in 25% of patients. In multivariable analysis, higher TC levels were associated with increased odds of high nodal burden (OR = 1.56 per 1 mmol/L increase; 95% CI: 1.07–2.38; *P* = 0.026), whereas no statistically significant association was observed in univariate analysis. The optimism-corrected C-index was 0.61, indicating limited discriminative performance. Likelihood ratio testing and AIC comparison showed that inclusion of tumor size did not improve model fit (*χ*² = 0.17, *P* = 0.679,AIC 112.67 vs. 110.84). Sensitivity analysis based on TC quartiles did not demonstrate statistically significant differences, and no clear dose–response relationship was observed.

**Conclusion:**

Preoperative total cholesterol was observed to be associated with axillary nodal burden in breast cancer; however, given the limited sample size, low event rate, absence of key confounders, and poor model performance, these findings should be considered exploratory. The proposed model demonstrates limited discriminative ability and is not suitable for clinical application. Further validation in larger, well-designed studies is required.

## 1 Introduction

Breast cancer is the most frequently diagnosed malignancy among women worldwide and remains a leading cause of cancer-related mortality (1). According to GLOBOCAN 2022 estimates, approximately 2.3 million new cases and 670,000 deaths occurred globally (2). Despite significant advances in systemic therapy, axillary lymph node (ALN) status continues to be one of the most important prognostic factors in early-stage breast cancer (3).

In particular, a high axillary lymph node burden—commonly defined as more than four metastatic lymph nodes (pN2–N3 stage)—represents a critical threshold in clinical decision-making (4). Patients with high ALN burden are more likely to receive post-mastectomy radiotherapy and intensified systemic treatment (5). Therefore, identifying reliable preoperative indicators associated with high nodal burden remains of substantial clinical interest.

Metabolic reprogramming has been recognized as a hallmark of cancer (6). Cholesterol, a key structural component of cell membranes and lipid rafts, plays an essential role in signal transduction pathways involved in cell proliferation and migration (7). Moreover, cholesterol-derived metabolites such as 27-hydroxycholesterol have been shown to function as endogenous selective estrogen receptor modulators, potentially promoting tumor progression and metastasis (8), While obesity and dyslipidemia have been associated with breast cancer outcomes (9), the specific relationship between preoperative serum total cholesterol (TC) and the extent of nodal involvement has not been well established (10).

Most previous studies have focused on the binary presence of nodal metastasis (N0 vs. N+), rather than distinguishing between low and high nodal burden (11). Given the clinical implications of high ALN burden, therefore, we aimed to investigate whether preoperative serum TC levels are independently associated with increased odds of high ALN burden and to develop and internally validate a clinically applicable prediction model for individualized preoperative risk estimation.

## 2 Methods

### 2.1 Study design and patient population

This retrospective study included female patients with histologically confirmed invasive breast carcinoma who underwent modified radical mastectomy at a single tertiary center between January 2024 and December 2025. Patients who received neoadjuvant chemotherapy, endocrine therapy, or targeted therapy prior to surgery were excluded to avoid treatment-related alterations in nodal status.

Inclusion criteria were:

1. Primary operable breast cancer
2. Underwent upfront modified radical mastectomy
3. Available preoperative fasting serum lipid measurements
4. Complete pathological evaluation of axillary lymph nodes

Patients with distant metastasis at diagnosis or incomplete clinical data were excluded.

After screening, 100 patients with complete data on total cholesterol, tumor size, and nodal status were included in the final analysis.

### 2.2 Ethics statement

This retrospective study was approved by the Medical Ethics Committee of Hunan Cancer Hospital (Approval No. 2026 Scientific Research Expedited Review [79]). All methods were performed in accordance with the relevant guidelines and regulations and with the Declaration of Helsinki. The requirement for informed consent was waived by the Ethics Committee due to the retrospective design of the study.

### 2.3 Data collection and definitions

Clinicopathological data were extracted from medical records, including age at diagnosis, pathological tumor size, and number of metastatic lymph nodes.

High axillary lymph node burden was defined as the presence of more than four metastatic lymph nodes, consistent with pN2–N3 staging according to the AJCC 8th edition (12).

Preoperative fasting blood samples were obtained within one week prior to surgery. Serum total cholesterol (TC) levels were measured using an automated biochemical analyzer in the hospital central laboratory.

### 2.4 Statistical analysis

Continuous variables were summarized as mean ± standard deviation or median (interquartile range), as appropriate (13). Logistic regression analysis was performed to evaluate the association between preoperative TC levels and high axillary lymph node burden. TC was analyzed as a continuous variable (per 1 mmol/L increase). Multivariable logistic regression was conducted adjusting for pathological tumor size. Odds ratios (ORs) with 95% confidence intervals (CIs) were reported.

Model discrimination was assessed using receiver operating characteristic (ROC) analysis, and the concordance index (C-index) was calculated. Internal validation was performed using bootstrap resampling with 1000 iterations to estimate optimism-corrected performance and shrinkage. Calibration was evaluated using bootstrap-based calibration plots and mean absolute error.

A nomogram was constructed based on the final multivariable model to facilitate individualized risk estimation.

All statistical analyses were performed using R software (version 4.5.0). A two-sided P value < 0.05 was considered statistically significant.

## 3 Results

### 3.1 Patient characteristics

A total of 100 patients were included in the final analysis. The incidence of high axillary lymph node (ALN) burden (>4 positive nodes) was 25.0% (25/100).

The mean preoperative serum total cholesterol (TC) level was 5.51 ± 1.21 mmol/L. The median pathologic tumor size was 2.5 cm (IQR: 2.0–3.2 cm).

Baseline clinicopathological characteristics stratified by nodal burden status are summarized in Table 1.

**Table 1:** Baseline clinicopathological characteristics stratified by axillary lymph node burden.

Characteristic	Overall (*n* = 100)	Low burden (≤4 nodes), *n* = 75	High burden (>4 nodes), *n* = 25	*P* value
Total cholesterol (mmol/L), mean (SD)	5.51 (1.21)	5.35 (1.03)	6.02 (1.55)	0.015
Tumor size (cm), mean (SD)	2.69 (0.95)	2.65 (0.97)	2.79 (0.89)	0.530

Data are presented as mean (SD). Continuous variables were compared using Student’s t-test.

### 3.2 Association between serum TC and high ALN burden

In univariate logistic regression analysis, preoperative serum TC level was positively associated with increased odds of high ALN burden, although statistical significance was not reached (OR = 1.36 per 1 mmol/L increase, *P* = 0.148).

After adjustment for pathological tumor size, multivariate logistic regression analysis demonstrated that higher preoperative TC levels were significantly associated with an increased risk of high ALN burden (OR = 1.56 per 1 mmol/L increase;95% CI: 1.07–2.38;*P* = 0.026).

Pathological tumor size was not significantly associated with high nodal burden in the adjusted model (OR = 1.11;95% CI: 0.67–1.81;*P* = 0.678).

Detailed regression results are presented in Table 2.

**Table 2:** Multivariate logistic regression analysis for high axillary lymph node burden.

Variable	OR (95% CI)	*P* value
Total cholesterol (per 1 mmol/L increase)	1.56 (1.07–2.38)	0.026
Tumor size (cm)	1.11 (0.67–1.81)	0.678

Adjusted for pathological tumor size.

To further evaluate the contribution of tumor size to the predictive model, we performed a likelihood ratio test (LRT) comparing the model including total cholesterol (TC) alone with the model including both TC and tumor size. The LRT showed no significant improvement in model fit (*χ*² = 0.17, *P* = 0.679).Consistently, AIC comparison demonstrated that the TC + tumor size model had a slightly higher AIC than the TC-only model (112.67 vs. 110.84), suggesting no improvement in predictive performance.These findings indicate that tumor size contributed minimally to the model in this dataset, supporting a parsimonious model including TC alone.

### 3.3 Model discrimination and validation

The apparent C-index of the multivariable logistic regression model was 0.64. Internal validation using 1000 bootstrap resamples yielded an optimism-corrected C-index of 0.61, This indicates that the model has a lower ability to discriminate between high and low axillary lymph node burden. The shrinkage slope after bootstrap correction was 0.85, suggesting limited overfitting.

**Figure 1:**
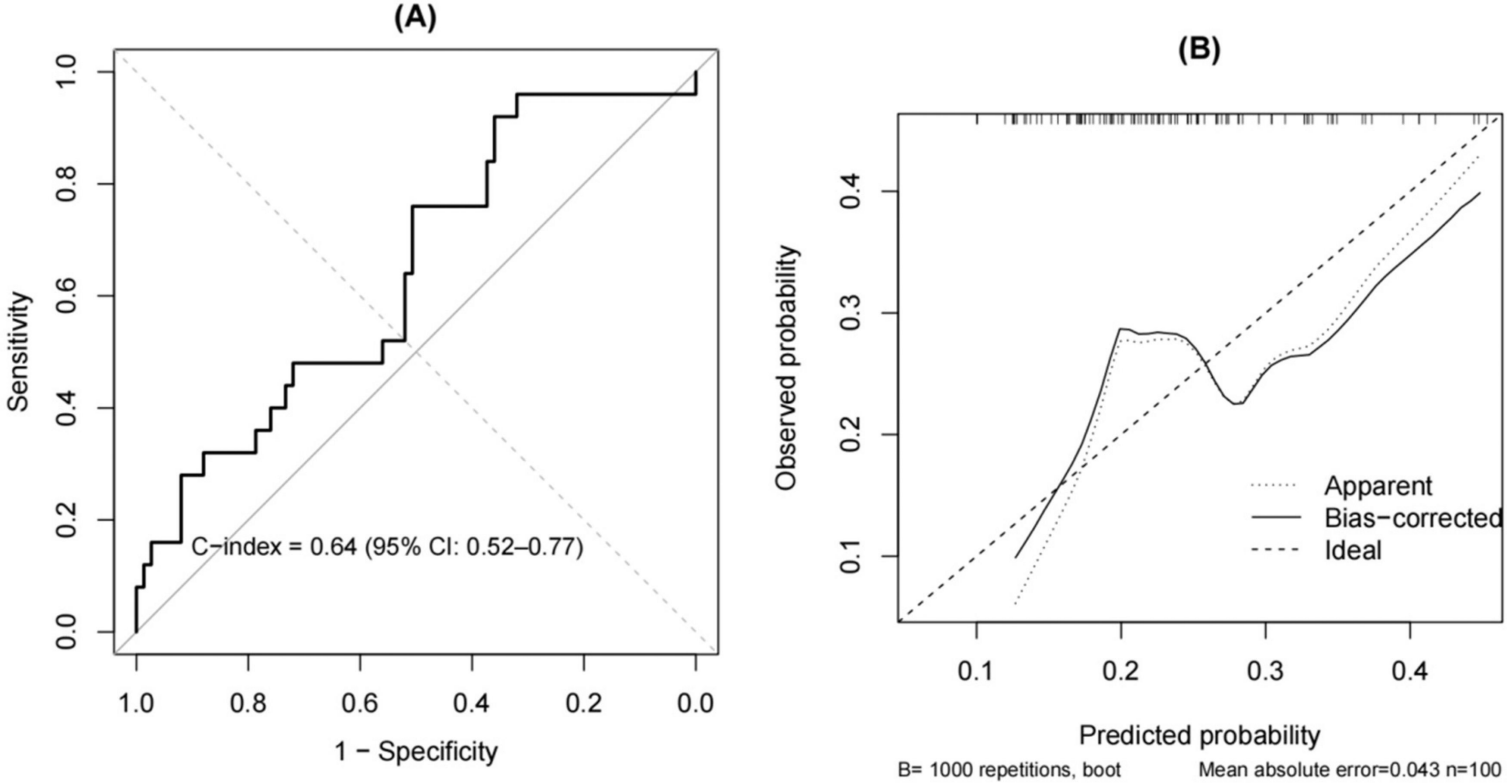
Discrimination and calibration performance of the multivariable model for predicting high axillary lymph node burden. **(A)** Receiver operating characteristic (ROC) curve of the model incorporating preoperative total cholesterol and tumor size. The apparent C-index was 0.64 (95% CI: 0.52–0.77). **(B)** Calibration plot based on 1000 bootstrap resamples demonstrating good agreement between predicted and observed probabilities (mean absolute erro*r* = 0.044).

Calibration analysis demonstrated good agreement between predicted and observed probabilities across risk strata. The mean absolute error between predicted and observed outcomes was 0.044, supporting satisfactory calibration performance (Figure 1).

### 3.4 Nomogram development

**Figure 2:**
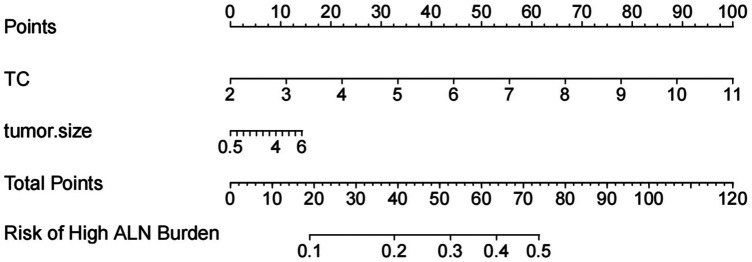
Nomogram for individualized prediction of high axillary lymph node burden.

Based on the multivariable logistic regression model incorporating preoperative total cholesterol and pathological tumor size, a nomogram was constructed to facilitate individualized preoperative risk estimation ((Figure 2).

Each predictor was assigned a weighted score proportional to its regression coefficient. By summing the total points corresponding to individual patient characteristics, the estimated probability of high axillary lymph node burden can be obtained.

For example, a patient with a preoperative TC level of 6.5 mmol/L and a tumor size of 3 cm would correspond to an estimated 33.4% probability of high ALN burden according to the nomogram. The nomogram thus provides a visual and clinically applicable tool for individualized risk assessment.

The nomogram was constructed based on a multivariable logistic regression model incorporating preoperative total cholesterol (TC) and pathological tumor size. Points assigned to each predictor are summed to obtain total points, which correspond to the estimated probability of high ALN burden.

### 3.5 Sensitivity analysis

To assess the robustness of the findings, serum TC levels were further categorized into quartiles. Compared with patients in the lowest quartile (Q1), those in higher quartiles demonstrated progressively increased odds of high ALN burden [Q2: OR = 1.24 (95% CI: 0.29–5.65); Q3: OR = 2.14 (95% CI: 0.55–9.35); Q4: OR = 2.86 (95% CI: 0.77–12.23)], after adjustment for tumor size.

Although point estimates increased across quartiles, the associations did not reach statistical significance, and no clear dose–response relationship can be established based on the present data. The corresponding forest plot is provided in Supplementary Figure S1.

## 4 Discussion

In this retrospective study, we observed a potential association between preoperative serum total cholesterol (TC) and high axillary lymph node (ALN) burden in patients with breast cancer. A preliminary model incorporating TC and tumor size was constructed; however, its overall performance was limited and should be interpreted with caution.

A notable finding was the discrepancy between univariate and multivariable analyses. TC was not statistically significant in univariate analysis but became significant after adjustment for tumor size. This pattern may reflect residual confounding or a suppression effect; however, it may also arise from statistical instability related to the relatively small sample size and limited number of events (14). Notably, tumor size itself was not significantly associated with nodal burden in the multivariable model, and likelihood ratio testing and AIC comparison indicated that its inclusion did not improve model fit. Therefore, the observed association between TC and nodal burden should not be interpreted as robust evidence of an independent effect.

From a biological perspective, cholesterol metabolism has been implicated in tumor progression through its roles in membrane biosynthesis, steroid hormone production, and intracellular signaling (15). Experimental evidence suggests that cholesterol and its metabolites could influence tumor cell proliferation, migration and invasion (16) via pathways such as PI3 K/AKT and MAPK signaling (17). Cholesterol-derived metabolites, such as 27-hydroxycholesterol, have been reported to interact with estrogen receptor signaling (18) and may potentially influence tumor-related biological processes in breast cancer (19). However, these findings are primarily derived from experimental studies, and the present observational analysis cannot establish any causal relationship. Therefore, such mechanistic considerations should be interpreted as speculative and hypothesis-generating.

Lymph node metastasis represents a key step in breast cancer progression and reflects complex interactions between tumor biology and the host microenvironment (20, 21). Although lipid metabolism may play a role in shaping the tumor microenvironment (22, 23), the association observed in this study remains exploratory and may be influenced by unmeasured confounding factors.

Importantly, the discriminative performance of the proposed model was limited. The optimism-corrected C-index of 0.61 indicates poor ability to distinguish between patients with and without high nodal burden, only marginally better than chance. Although calibration appeared acceptable, discrimination is a critical requirement for clinical utility. Therefore, the current model is not suitable for clinical application and should not be used to guide treatment decisions.

Given the limited number of events (*n* = 25) relative to the number of predictors, the events-per-variable ratio was borderline, which may lead to coefficient instability, overfitting, and potential inflation of effect size estimates. Although bootstrap internal validation was performed, it cannot fully compensate for the limitations imposed by low statistical power. These factors further support the exploratory nature of the model (24).

Several limitations of this study should be acknowledged. First, this was a single-center retrospective study with a relatively small sample size and a limited number of events, which may limit the generalizability of the findings (25). Second, important confounding variables—including body mass index, menopausal status, hormone receptor status, HER2 status, tumor grade, lymphovascular invasion, and statin use—were not included in the analysis. The omission of these factors may introduce residual confounding and limits the validity of the observed associations (26). Third, the retrospective design precludes causal inference and may be subject to selection bias (27). Fourth, serum TC was measured at a single time point, and potential temporal variations were not assessed (28). Finally, although internal validation was performed, external validation in independent cohorts is required before any further consideration of clinical applicability.

In addition, sensitivity analysis based on TC quartiles did not demonstrate statistically significant differences. Although point estimates increased across quartiles, the trend did not reach statistical significance, and no clear dose–response relationship can be established based on the present data.

Future studies with larger, multicenter cohorts and more comprehensive variable adjustment are needed to validate these findings. The integration of metabolic markers with molecular, imaging, and clinical features may further improve predictive performance (29) and provide deeper insights into the role of lipid metabolism in breast cancer progression (30).

## 5 Conclusion

This study suggests a potential association between preoperative serum total cholesterol and axillary lymph node burden in breast cancer. However, due to the limited sample size, low event rate, and lack of adjustment for important confounding variables, the findings should be interpreted with caution.

The proposed model is exploratory in nature and is not suitable for clinical application at this stage. Further validation in larger, multicenter cohorts with more comprehensive variable adjustment is required before any clinical use can be considered (31).

## Authors’ contributions

Xingxing Ouyang and Lisha Wu contributed equally to this work. Xingxing Ouyang and Lisha Wu participated in study conception, data collection, and manuscript drafting. Yanhua Zhou supervised the study, interpreted the data, and critically revised the manuscript. All authors contributed to the article and approved the submitted version.

## Data Availability

The original contributions presented in the study are included in the article/Supplementary Material, further inquiries can be directed to the corresponding author.
