# Supplementary material for: Preoperative total cholesterol and axillary lymph node burden in breast cancer: an exploratory analysis with a preliminary nomogram
Source: Front Surg. 2026 Jul 1;13:1860780. doi: 10.3389/fsurg.2026.1860780 (PMC13369522; doi:10.3389/fsurg.2026.1860780)
Supplement: Supplementary file 1 [file Image1.pdf]

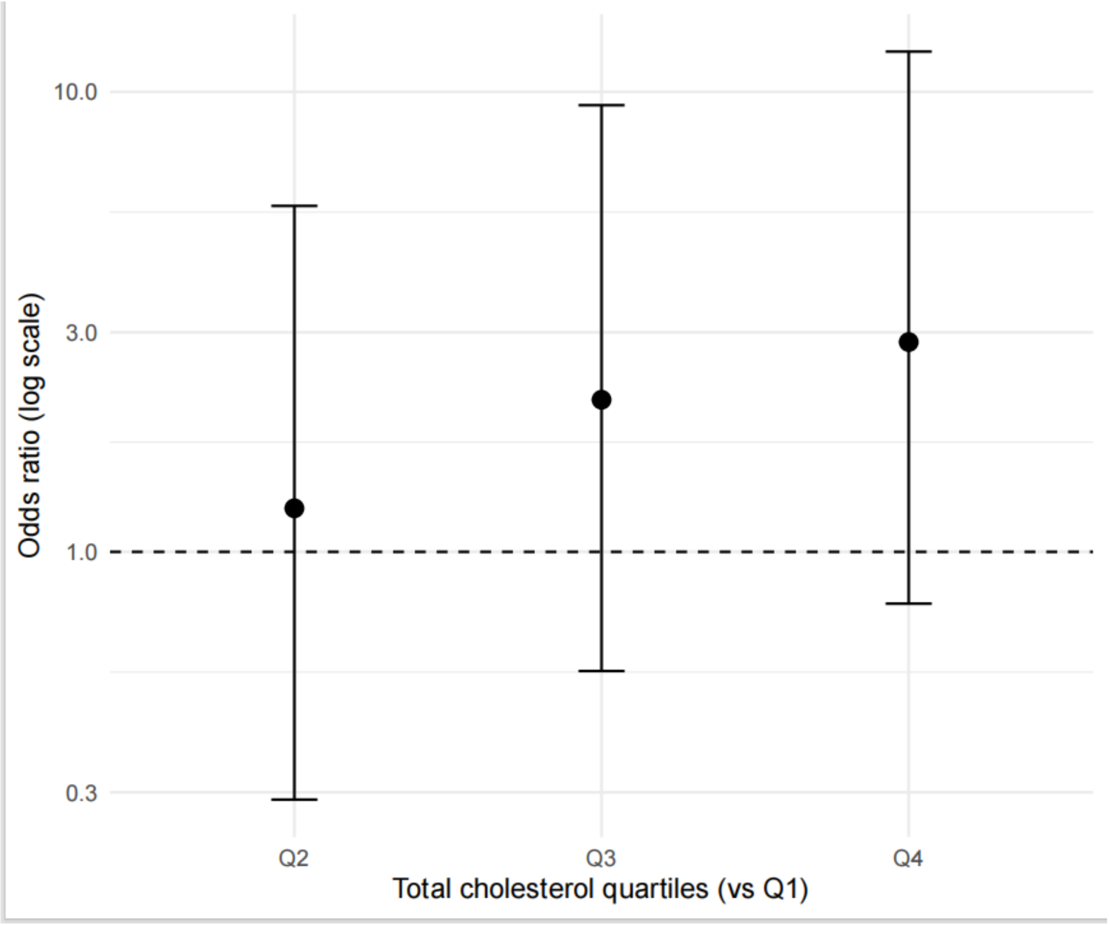

Supplementary Figure S1. Association between total cholesterol quartiles and high axillary lymph node burden.
Odds ratios were adjusted for pathological tumor size. The lowest quartile (Q1) was used as the reference group. Error bars represent 95% confidence intervals. A positive dose–response trend was observed across increasing TC quartiles (P for trend = 0.085).
